# Occurrence and significance of pathogenicity and fitness islands in environmental vibrios

**DOI:** 10.1186/s13568-018-0704-2

**Published:** 2018-10-30

**Authors:** Savannah Klein, Shannon Pipes, Charles R. Lovell

**Affiliations:** Department of Biological Sciences, University of South Carolina, 715 Sumter St, Room 401, Columbia, SC 29208 USA

**Keywords:** *Vibrio parahaemolyticus*, *Vibrio vulnificus*, Pathogenicity islands

## Abstract

Pathogenicity islands (PAIs) are large genomic regions that contain virulence genes, which aid pathogens in establishing infections. While PAIs in clinical strains (strains isolated from a human infection) are well-studied, less is known about the occurrence of PAIs in strains isolated from the environment. In this study we describe three PAIs found in environmental *Vibrio vulnificus* and *Vibrio parahaemolyticus* strains, as well as a genomic fitness island found in a *Vibrio diabolicus* strain. All four islands had markedly different GC profiles than the rest of the genome, indicating that all of these islands were acquired via lateral gene transfer. Genes on the PAIs and fitness island were characterized. The PAI found in *V. parahaemolyticus* contained the *tdh* gene, a collagenase gene, and genes involved in the type 3 secretion system II (T3SS2). A *V. vulnificus* environmental strain contained two PAIs, a small 25 kbp PAI and a larger 143 kbp PAI. Both PAIs contained virulence genes. Toxin–antitoxin (TA) genes were found in all three species: on the *V. diabolicus* fitness island, and on the *V. parahaemolyticus* and *V. vulnificus* PAIs.

## Introduction

*Vibrio parahaemolyticus* and *Vibrio vulnificus* can cause illnesses in humans, with an estimated 80,000 cases occurring annually in the United States (Scallan et al. 2011; CDC 2017). The hospitalization and mortality rates of *V. parahaemolyticus* gastroenteritis are 22% and 1%, respectively (Scallan et al. 2011). Although cases are usually mild and tend to resolve themselves after 1–3 days, *V. parahaemolyticus* is responsible for the majority of vibriosis cases (Scallan et al. 2011). *V. vulnificus* cases are less common; only about 100 occur each year in the United States. However, the hospitalization and mortality rates of this bacterium are much higher, at 92% and 35%, respectively (Scallan et al. 2011). *V. vulnificus* also causes sepsis and necrotizing fasciitis if it enters the body through an open wound. The majority of reported *V. vulnificus* cases are from wound infections (45%) and septicemia (43%); only 5% are gastroenteritis (Scallan et al. 2011). The mortality rate of *V. vulnificus* when it invades the bloodstream (sepsis) increases to 60%. Pathogenesis of both species is complex, and while some virulence factor genes have been implicated, the mechanisms underlying *V. vulnificus* and *V. parahaemolyticus* virulence are not well understood (Broberg et al. 2011; Lovell 2017; Klein and Lovell 2016).

Pathogenicity islands (PAIs), a subgroup of genomic islands that aid in and contribute to pathogenesis, have been found in clinical strains of both *V. vulnificus* and *V. parahaemolyticus*. PAIs are large chromosomal regions that are flanked by tRNA genes, and are usually associated with mobile genetic elements, such as phage, plasmid, integron, and transposon genes. A genomic island must contain at least one virulence gene, or gene that contributes to pathogenesis, to be considered a PAI. The size of PAIs ranges from 10 to 200 kbp (Schmidt and Hensel 2004; Hacker and Kaper 2000; Hacker and Carniel 2000) and the average *Vibrio* genome is 4.5 mbp (Pipes et al. in preparation), meaning that a single PAI could make up as much as 4% of a *Vibrio* genome. PAIs are flanked by highly conserved tRNA genes that act as both integration and excision sites. The majority (~ 75%) of PAIs discovered have tRNA flanking sequences (Schmidt and Hensel 2004; Hacker and Kaper 2000). Additionally, tRNA loci are often found on extrachromosomal elements, such as plasmids and bacteriophages. This indicates that the most likely mechanism for extrachromosomal element insertion is homologous recombination between the extrachromosomal element tRNA and PAI flanking tRNA loci (Hacker and Kaper 2000).

There is considerable evidence that PAIs are acquired horizontally via one or more lateral transfer events. Within some PAIs there is evidence of one large transfer event, while other PAIs are more “mosaic-like.” The “mosaic-like” composition of certain PAIs is caused by multiple, independent lateral transfer events (Hacker and Kaper 2000; Schmidt and Hensel 2004). PAIs usually differ in codon usage biases and have a markedly lower or higher GC content than the rest of the genome (Schmidt and Hensel 2004; Hacker and Kaper 2000; Hacker et al. 1997; Hacker and Carniel 2000). This supports the idea that recognizable PAIs are incorporated into a genome via lateral gene transfer from a dissimilar or unrelated organism (donor) having differing GC content and codon usage than the recipient (Schmidt and Hensel 2004). However, PAI GC content may not differ from that of the core genome if the donor and recipient microorganisms are closely related (Hacker and Kaper 2000). Dissimilarities in base composition confirm that detectable lateral transfer of PAIs must have been of recent origin, as insufficient time for genetic drift has passed (Schmidt and Hensel 2004).

PAIs have been found in clinical strains of *V. vulnificus* and *V. parahaemolyticus* (e.g. Makino et al. 2003; Wang et al. 2006; Sugiyama et al. 2008; Quirke et al. 2006; Cohen et al. 2007). Nine PAIs have been identified in *V. parahaemolyticus*, with VPAI-1 and VPAI-7 (*V. parahaemolyticus* pathogenicity island one and *V. parahaemolyticus* pathogenicity island seven) being the most studied (Ceccarelli et al. 2013). VPAI-1 is a 22 kbp island that is found on chromosome 1 in some strains, and chromosome 2 in others (Wang et al. 2006; Chen et al. 2011). This observation provides evidence for the mobility of this genomic island. VPAI-7 is the largest *Vibrio* genomic island found to date. This island contains the virulence factors TDH (thermostable direct hemolysin) and type III secretion system 2 (T3SS2) (Makino et al. 2003; Sugiyama et al. 2008). Other names for VPAI-7 include VPaIα or *tdh*VPA (Xu et al. 2017) and parts of VPAI-7 have been found in other *Vibrio* species, such as *Vibrio mimicus* (Gennari et al. 2011).

Genomic islands have been found in *V. vulnificus* clinical strains YJ016 and CMCP6, with 14 regions ranging in size from 14 to 117 kpb. A superintergon (SI) and nine *V. vulnificus* genomic islands (VVI–I to VVI–IX) have been found in these clinical strains. PAIs have not been detected in environmentally derived *V. vulnificus* strains (Quirke et al. 2006). *V. vulnificus* VVI–I has been found in the *Vibrio cholerae* biotype El Tor and O139 serogroup. The functional role of this island has not been determined but its presence in *V. cholerae* supports the idea that these regions can be transferred to other closely related species (O’Shea et al. 2004).

Work on *Vibrio* PAIs is heavily skewed toward clinical strains, with the pathogenic potential of naturally-occurring (environmental) strains rarely considered. In this study, we characterized four genomic islands found in environmental *Vibrio* strains: a PAI within a *V. parahaemolyticus* strain, two novel PAIs within a *V. vulnificus* strain, and a novel fitness island found in a *Vibrio diabolicus* strain. Environmental *Vibrio* strains, and the PAIs within them, could serve as reservoirs for virulence genes.

## Materials and methods

### Strain isolation

Environmental *V. parahaemolyticus* and *V. diabolicus* strains were isolated previously (Gutierrez West et al. 2013; Klein et al. 2014) from the pristine North Inlet salt marsh estuary near Georgetown, SC, USA (33°20′N, 79°12′W). Environmental *V. vulnificus* strains were also isolated near Georgetown, SC; however, they were isolated from lower salinity waters in Winyah Bay and the Waccamaw River. Water samples were plated on CHROMagar *Vibrio* (DRG International, NJ, USA) for isolation of *V. vulnificus* strains following the US Food and Drug Administration protocol (DePaola and Kaysner 2004). *Vibrio* strains were routinely cultivated on saline Luria Agar (SLA; per L; 10 g tryptone, 5 g yeast extract, 27 g NaCl, 15 g Bacto Agar). *V. parahaemolyticus* TS-8-11-4 and *V. diabolicus* JBS-8-11-1 were deposited into the DSMZ Public Culture Collection and were assigned their respective accession numbers: DSM 107522 and DSM 107521.

### Whole genome sequencing

Genomic DNA was isolated through the Wizard Genomic DNA Purification kit following the protocol for Gram negative organisms (Promega, Madison, WI, USA**)**. After DNA was extracted, DNA quantity was measured via Quibit fluorimetry. Libraries were prepared and then sequenced using an Illumina MiSeq (V3 26300 base) at the Indiana University Center for Genomic Studies as a part of the Genome Consortium for Active Teaching NextGenSequencing Group (GCAT-SEEK) shared run (Buonaccorsi et al. 2011, 2014). Sequencing reads were filtered (median phred score 0.20), trimmed (phred score 0.16), and assembled using the paired-end de novo assembly option in NextGENe V2.3.4.2 (SoftGenetics, State College, PA, USA). The assembled genomes were uploaded to the Rapid Annotation with Subsystem Technology (RAST) web service (Aziz et al. 2008; Overbeek et al. 2005, 2014) for analysis, guided contig reordering and assembly improvement. Genomes were aligned based on completed sequences using dotplot comparisons. Whole genome sequence data obtained from this work were submitted to the NCBI GenBank and assigned the accession numbers: PKQA00000000, PKPY00000000, and PKPZ00000000.

### PAI detection and characterization

The fully sequenced genomes were uploaded to TUBIC (Tiajin University Bioinformatics Center) to determine their GC profiles (http://tubic.tju.edu.cn/). This tool displays GC content variation across a genome and can be useful for identifying genomic regions that differ from the rest of the genome in GC content (Gao and Zhang 2006). Genomic islands that were detected via TUBIC were isolated and the island nucleotide sequence was uploaded to RAST to identify and characterize the specific genes found on the genomic islands (http://rast.nmpdr.org/). NCBI GenBank was also used to characterize genomic island genes (http://www.ncbi.nlm.nih.gov/genbank/). Gene sequences of interest were edited and maximum-likelihood trees were constructed using the Kimura 2-parameter model with Mega version 7. DNAPlotter was used to visualize the circular chromosomes of the *Vibrio* strains (Carver et al. 2009).

## Results

### *V. parahaemolyticus* island

**Fig. 1:**
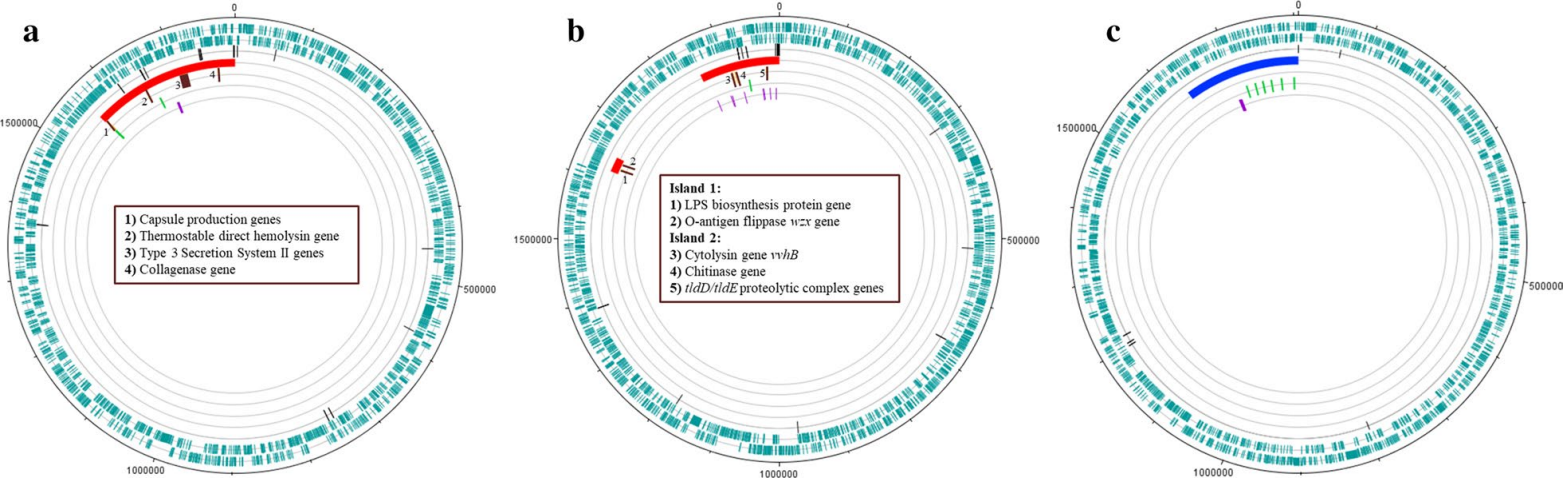
**a**–**c** Circular presentation of the second chromosome of **a** *Vibrio parahaemolyticus* environmental strain TS-8-11-4, **b** *Vibrio vulnificus* environmental strain WR-2-BW, and **c** *Vibrio diabolicus* environmental strain JBS-8-11-1. Track 1, forward coding sequences; track 2, reverse coding sequences; track 3, tRNA genes; track 4, red, pathogenicity islands, blue, genomic fitness islands; track 5, virulence and virulence-associated genes; track 6, genes involved in toxin–antitoxin systems; track 7, mobile genetic elements. Virulence and virulence-associated genes are numbered and are defined via the center text boxes

*Vibrio parahaemolyticus* strain TS-8-11-4 was isolated from salt marsh sediments (Gutierrez West et al. 2013; Klein et al. 2014) at the pristine North Inlet estuary in South Carolina, USA. This strain had a genome of 4.98 mbp; chromosome 1 was 3.19 mbp in length and chromosome 2 was 1.78 mbp in length. The majority of the genome contained a GC content of 45.57%, which is typical for *V. parahaemolyticus* (Farmer and Janda 2005). However, this strain contained a 223 kbp island that had a markedly lower GC content (41.5%) not typical of *V. parahaemolyticus*. The majority (69%) of genes on the TS-8-11-4 PAI could not be assigned specific identities and were thus designated hypothetical. The genomic island of *V. parahaemolyticus* TS 8-11-4 was on the second chromosome of and it harbored virulence genes (Fig. 1a). The virulence factor genes that were found on this island included the thermostable direct hemolysin gene, genes involved in the type three secretion system II (T3SS2), a collagenase gene, as well as capsule production genes.

### *V. vulnificus* islands

*Vibrio vulnificus* strain WR-2-BW was isolated near Georgetown, SC from Waccamaw River waters. Its genome (4.96 mpb) contained two chromosomes, the first chromosome larger (2.96 mbp) than the second (1.99 mbp). The average GC content of *V. vulnificus* ranges from 46 to 48% (Farmer and Janda 2005), and the average GC content of strain WR-2-BW was 46.83%. Two regions within the genome had GC contents that were markedly lower from the rest of the genome. The first region had a GC content of 38.2% and the second region had a GC content of 42.5%; both of which are lower than the typical GC content of *V. vulnificus* strains. These regions were found on the second chromosome (Fig. 1b). The first region was a 25 kbp island and the second region was a 143 kbp island. The 25 kbp island was 30 genes in length, and had two genes that had virulence-related functions, which include a putative LPS biosynthesis protein gene and an O-antigen flippase *wzx* gene. The 143 kbp island contained the cytolysin gene *vvhB*, a chitinase gene, *tldD*/*tldE* proteolytic complex genes, and Type IV secretory pathway components. The 143 kbp genomic island was comprised of 160 genes in total, 63% of which were characterized as hypothetical or had unknown function.

### *V. diabolicus* island

*Vibrio diabolicus* strain JBS-8-11-1 was isolated previously from North Inlet salt marsh sediments (Gutierrez West et al. 2013; Klein et al. 2014). Its genome (5.04 mbp) was comprised of two chromosomes, the first (3.23 mbp) being larger than the second (1.81 mbp). Its GC content was typical of other *V. diabolicus* genomes (44.91%) (Goudenege et al. 2014), except for a 182 kbp island, located on chromosome 2, which had a GC content of 40.8%. Eighty-two percentage of the island consisted of hypothetical genes. This island harbored no known virulence genes; it is hereafter referred to as a fitness island (Fig. 1c). Three genes, a phage DNA synthesis gene, a phage DNA replication gene, and a gene encoding a phage capsid protein, were located very close to each other on the fitness island. Thirteen genes involved in toxin–antitoxin (TA) systems were located on the fitness island.

## Discussion

The genomic island of *V. parahaemolyticus* TS 8-11-4 was deemed a PAI due to the presence of virulence genes on this island, despite its environmental origin (Schmidt and Hensel 2004; Hasan et al. 2010; Dobrindt et al. 2004). The thermostable direct hemolysin gene (*tdh*) was found on this island, as well as genes involved in the type three secretion system II (T3SS2). Both the *tdh* gene and T3SS2 complex are the two major virulence factors implicated in *V. parahaemolyticus* pathogenesis (Makino et al. 2003; Park et al. 2004; Yanagihara et al. 2010). A collagenase gene was found on the island; collagenase is thought to be involved in *V. parahaemolyticus* virulence (Gode-Portratz et al. 2011). The genomic island of *V. parahaemolyticus* strain TS-8-11-4 is a PAI, and more specifically, because it contains *tdh* and T3SS2 genes, we designate this island as a VPAI-7 (VPaIα or *tdh*VPA) (Makino et al. 2003; Sugiyama et al. 2008; Xu et al. 2017).

Four genes involved in capsule production, as well as one integrase gene, and a Na^+^/H^+^ antiporter (*nhaA*) were also found on this PAI. Capsules aid pathogens in evasion of host immune defenses, establishing infections, and survival in harsh environments, such as the stomach. *V. parahaemolyticus* virulence is correlated with capsule production (Broberg et al. 2011; Letchumanan et al. 2014). One capsule gene had high homology with Gram positive capsule production genes. This is interesting because vibrios are Gram negative organisms, so this gene may have been acquired laterally. An integrase gene was found near the center of the island. Integrase genes are associated with PAIs and function to integrate foreign DNA into the genome (Hacker and Kaper 2000). Usually VPAI-7 does not contain an integrase gene, but a few transposon genes instead (Ceccarelli et al. 2013). Finally, we determined that a *nhaA* gene is located on this genomic island. *nhaA* genes encode Na^+^/H^+^ antiporters, which transport ions to balance pH. Na^+^/H^+^ antiporters aid *V. cholerae* in environmental persistence (Vimont and Berche 2000) and are essential for *Yersinia pestis* virulence (Minato et al. 2013).

Similar to *V. parahaemolyticus*, the two islands found for the *V. vulnificus* WR-2-BW strain are characterized as PAIs due to the presence of virulence genes and virulence-related genes. Two of these genes had virulence-related functions, a putative LPS biosynthesis protein gene and an O-antigen flippase *wzx* gene. These genes are virulence-associated factors, as they do not directly cause host cell damage, but they do contribute to pathogenesis, aiding in the establishment of infections. Lipopolysaccharide (LPS) is a main component of the outer membrane of Gram negative bacteria, and is a known pyrogen (fever-producing agent) (McPherson et al. 1991; Jones and Oliver 2009). Phylogenies show that the LPS biosynthesis protein gene from *V. vulnificus* WR-2-BW was closely related to an LPS biosynthesis protein gene from a *Vibrio coralliilyticus* species. The O-antigen flippase *wzx* gene is part of the major class of O-antigen gene clusters, and it encodes a hydrophobic protein with 12 potential transmembrane segments (Liu et al. 1996).

A cytolysin secretion gene, *vvhB*, was found also found on the 143 kbp *V. vulnificus* island. Cytolysins lyse erythrocytes by forming small pores in the cytoplasmic membrane or binding to cholesterol to interrupt potassium and sodium ion channels (Choi et al. 2002). In *V. vulnificus*, the expression and mechanism of cytolysins *vvhA* and *vvhB* are not fully understood, however, they are both believed to play a role in pathogenicity (Choi et al. 2002). They are homologous to a known *V. cholerae* El Tor hemolysin (Choi et al. 2002; Yamamoto et al. 1990). Phylogenies show that the *vvhB* gene in the *V. vulnificus* WR-2-BW strain was 99% identical to other *V. vulnificus vvhB* genes from other strains.

Other genes of interest on the 143 kbp PAI include a chitinase gene, *tldD*/*tldE* proteolytic complex genes, and type IV secretory pathway components. In *Escherichia coli*, it was shown that the TldD and TldE proteins could be involved in regulating gyrase function as well as aiding in proteolytic activity (Allali et al. 2002). The chitinase gene had a 99% blast identity score to the chitinase gene found in the *V. vulnificus* YJ016 strain; however, the chitinase gene in YJ016 is located on the first chromosome and WR-2-BW’s chitinase gene is located in the second chromosome. Chitinous exoskeletal materials of invertebrates can be a source of carbon and nitrogen for bacteria; vibrios in particular have a well-known association with marine copepods (Kaneko and Colwell 1975; Lovell 2017). *V. cholerae* has a well-studied association with copepods, which commonly serve as a vector of cholera infections in Bangladesh water systems (Tamplin et al. 1990). Chitinase has been identified as part of the mechanism for adsorption and attachment to copepods, which relates to its ability to colonize its host and degrade the host exoskeleton, increasing the overall ecological fitness of the vibrios (Huq et al. 1983; Nalin et al. 1979; Bhowmick et al. 2006).

**Fig. 2:**
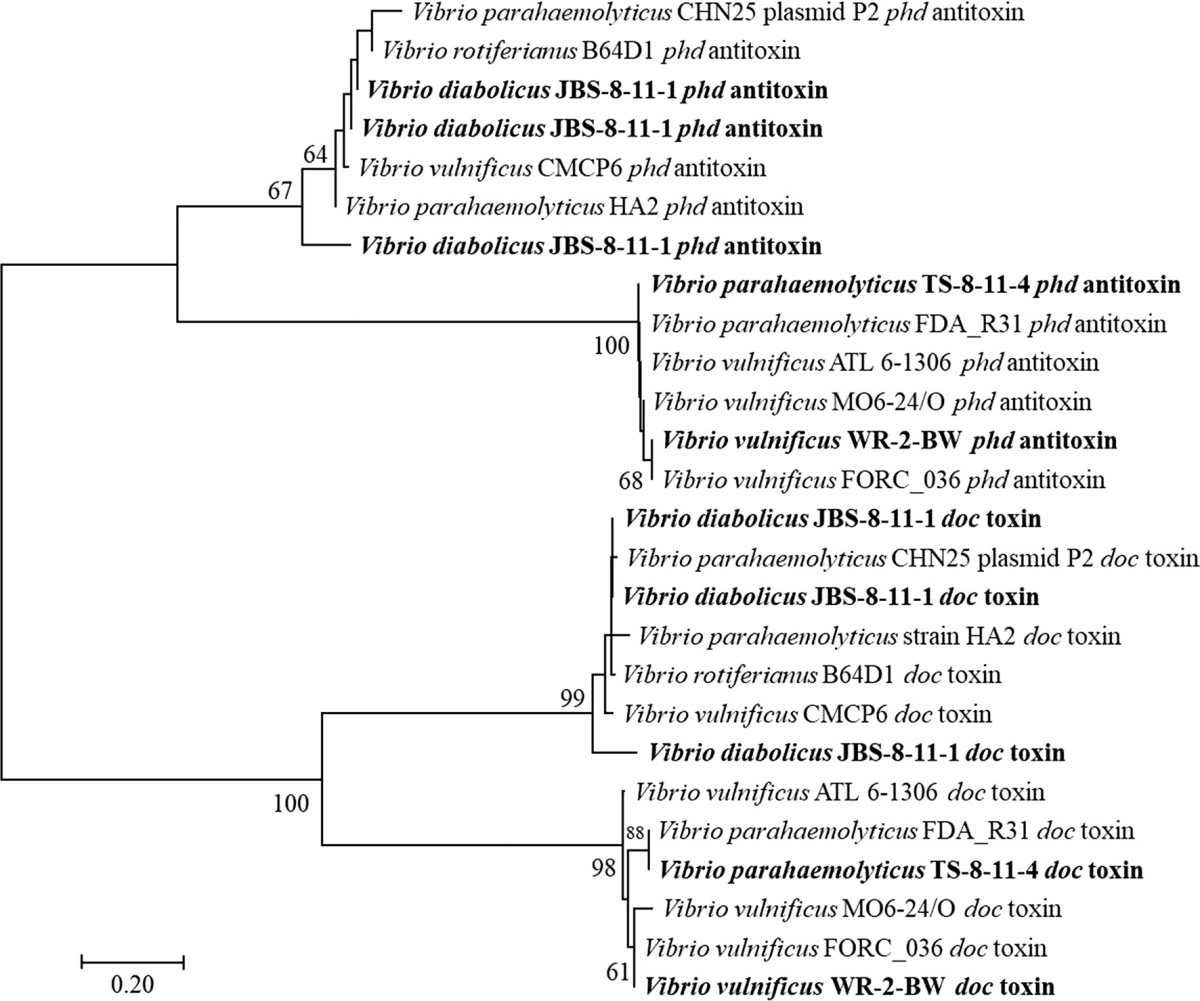
Maximum-likelihood phylogeny (Kimura 2-parameter model) of *doc* toxin genes and *phd* antitoxin genes. Bold indicates sequences obtained from this study. The bootstrap values represent 1000 replications, and values of less than 50 are not shown. The reference sequences were acquired from NCBI GenBank

*Vibrio diabolicus* had a large genomic island that did not contain any virulence factors or virulence associated genes, which we defined as a fitness island, as it contained genes that would aid the organism in persistence in the environment. Toxin–antitoxin (TA) systems are found either on plasmids, genomic islands, or within the chromosome and are made up of closely linked toxin and antitoxin genes. The encoded labile antitoxin protects the host from the stable toxin, while competitor cells that do not have the TA system (and respective antitoxin) are eliminated (Hayes 2003; Van Melderen and Saavedra 2009). Sometimes TA systems are referred to as “addiction modules” because the host cell is dependent on the antitoxin (Van Melderen and Saavedra 2009). The toxin and respective antitoxin loci are usually found neighboring each other, often overlapping (Hayes 2003). Seven type II TA toxins were found on JBS-8-11-1’s fitness island, along with their neighboring respective antitoxins. Type I TAs include RNA antitoxins, while type II TAs have protein antitoxins (Hayes 2003). The *relE*, *yafQ*, and *yoeB* toxin genes encode mRNA interferase endoribonucleases; all three of these toxin genes were detected on this fitness island. The *doc* toxin gene (death on curing) inhibits translation by blocking translation elongation at the 30S ribosomal subunit (Lui et al. 2008); three copies of the *doc* toxin gene and three copies of its antitoxin partner gene, *phd* (prevent host death) were found on JBS-8-11-1’s fitness island. *doc* toxin genes and *phd* antitoxin genes are widespread in vibrios and were also found on *V. parahaemolyticus* strain TS-8-11-4’s PAI as well as *V. vulnificus* strain WR-2-BW’s PAI (Fig. 2).

### Lateral gene transfer in environmental strains

PAIs are present in environmental *Vibrio* strains and are most likely acquired via lateral gene transfer. All four of the islands described here have significant lower GC content than the rest of the genome, providing evidence that these islands originated from a foreign source and were transferred into these genomes relatively recently. Additional evidence includes mobile genetic elements, such as phage and plasmid genes, integrases, and transposons. Virulence loci on VPAI-7 have been detected in environmental species that do not cause human infections: *Vibrio mimicus*, *Vibrio harveyi*, and *Vibrio natriegens* (Gennari et al. 2011; Klein et al. 2014). Clearly, lateral transfer of individual virulence loci and/or entire PAIs is occurring between and among environmental vibrios. It is well documented that *V. cholerae* enters a natural competency state in the presence of chitin or under low-nutrient conditions (Hazen et al. 2010; Metzger and Blokesch 2016); however, less is known about uptake of exogenous DNA by other *Vibrio* species. Further studies examining the rates of lateral transfer among vibrios in the environment are needed. Vibrios survive, persist and can undergo rapid population expansions (bloom) in coastal ecosystems. Consequently, the pathogenicity loci (and potential of said loci to be transferred laterally) of naturally occurring environmental strains are clearly important.

## Abbreviations

PAI: pathogenicity island
TA: toxin–antitoxin
LPS: lipopolysaccharide
T3SS2: type three secretion system II
*tdh*: thermostable direct hemolysin gene

## References

[CR1] Allali N, Afif H, Couturier M, Van Melderen L (2002). The highly conserved TldD and TldE proteins of *Escherichia coli* are involved in microcin B17 processing and in CcdA degradation. J Bacteriol.

[CR2] Aziz RK, Bartels D, Best AA, DeJongh M, Disz T (2008). The RAST server: rapid annotations using subsystems technology. BMC Genomics.

[CR3] Bhowmick R, Ghosal A, Chatterjee NS (2006). Effect of environmental factors on expression and activity of chitinase genes of vibrios with special reference to *Vibrio cholerae*. J Appl Micobiol.

[CR4] Broberg CA, Calder TJ, Orth K (2011). *Vibrio parahaemolyticus* cell biology and pathogenicity determinants. Microbes Infect.

[CR5] Buonaccorsi VP, Boyle MD, Grove D, Praul C (2011). GCATSEEKquence: genome consortium for active teaching of undergraduates through increased faculty access to next-generation sequencing data. CBE Life Sci Educ.

[CR6] Buonaccorsi VP, Peterson M, Lamendella G, Newman JD, Trun N (2014). Vision and change through the genome consortium for active teaching using next-generation sequencing (GCAT-SEEK). CBE Life Sci Educ.

[CR7] Carver T, Thomson N, Bleasby A, Berriman M, Parkhill J (2009). DNAPlotter: circular and linear interactive genome visualization. Bioinformatics.

[CR8] Ceccarelli D, Hasan NA, Huq A, Colwell RR (2013). Distribution and dynamics of epidemic and pandemic *Vibrio parahaemolyticus* virulence factors. Front Cell Infect Microbiol.

[CR9] Centers for Disease Control and Prevention (2017) *Vibrio* species causing vibriosis. Atlanta, GA. https://www.cdc.gov/vibrio/index.html

[CR10] Chen Y, Stine OC, Badger JH (2011). Comparative genomic analysis of *Vibrio parahaemolyticus*: serotype conversion and virulence. BMC Genomics.

[CR11] Choi HK, Park NY, Kim D, Chung HJ, Ryu S, Choi SH (2002). Promoter analysis and regulatory characteristics of vvhBA encoding cytolytic hemolysin of *Vibrio vulnificus*. J Biol Chem.

[CR12] Cohen AL, Oliver JD, DePaola A, Fiel EJ, Boyd EF (2007). Emergence of a virulent clade of *Vibrio vulnificus* and correlation with the presence of a 33-kilobase genomic island. Appl Environ Microbiol.

[CR13] DePaola A, Kaysner CA (2004) *Vibrio.* Bacteriological analytical manual online. U.S. Food and Drug Administration, Washington, DC. http://www.fda.gov/Food/FoodScienceResearch/LaboratoryMethods/ucm070830.htm

[CR14] Dobrindt U, Hochhut B, Hentschel U, Hacker J (2004). Genomic islands in pathogenic and environmental microorganisms. Nat Revi Microbiol.

[CR15] Farmer JJ, Janda JM, Brenner DJ, Krieg NR, Staley JT (2005). Family I. Vibrionaceae Véron, 1965, 5245^AL^. Bergey’s manual of systematic bacteriology.

[CR16] Gao F, Zhang C (2006). GC-Profile: a web-based tool for visualizing and analyzing the variation of GC content in genomic sequences. Nucleic Acids Res.

[CR17] Gennari M, Ghinidi V, Carbulutto G, Lleo MM (2011). Virulence genes and pathogenicity islands in environmental *Vibrio* strains nonpathogenic to humans. FEMS Microbiol Ecol.

[CR18] Gode-Portratz CJ, Kustusch RJ, Breheny PJ, Weiss DS, McCarter L (2011). Surfing sensing in *Vibrio parahaemolyticus* triggers a programme of gene expression that promotes colonization and virulence. Mol Microbiol.

[CR19] Goudenege D, Boursicot V, Versigny T, Bonnetot S, Ratiskol J, Sinquin C, LaPointe G, Roux F, Delbarre-Ladrat C (2014). Genome sequence of *Vibrio diabolicus* and identification of the exopolysaccharide HE800 biosynthesis locus. Appl Microbiol Biotechnol.

[CR20] Gutierrez West CK, Klein SL, Lovell CR (2013). The virulence factor genes *tdh*, *trh* and *tlh* occur at high frequency in *Vibrio parahaemolyticus* isolated from a pristine estuary. Appl Environ Microbiol.

[CR21] Hacker J, Carniel E (2000). Review: ecological fitness, genomic islands and bacterial pathogenicity: a darwinian view of the evolution of microbes. EMBO Rep.

[CR22] Hacker J, Kaper JB (2000). Pathogenicity islands and the evolution of microbes. Annual Rev Microbiol.

[CR23] Hacker J, Blum-Oehler G, Muldorfer I, Tschape H (1997). Pathogenicity islands of virulent bacteria: structure, function and impact on microbial evolution. Mol Microbiol.

[CR24] Hasan NA, Grim CJ, Haley BJ, Chun J, Alam M, Taviani E, Hoq M, Munk AC, Saunders E, Brettin TS, Bruce DC, Challocombe JF, Detter CJ, Han CS, Xie G, Nair B, Huq A, Colwell RR (2010). Comparative genomics of clinical and environmental *Vibrio mimicus*. PNAS.

[CR25] Hayes F (2003). Tonxins-antitoxins: plasmid maintenance, programmed cell death, and cell cycle arrest. Science.

[CR26] Hazen TH, Pan L, Gu J, Sobecky PA (2010). The contribution of mobile genetic elements to the evolution and ecology of vibrios. FEMS Microbiol Ecol.

[CR27] Huq A, Small EB, West PA, Huq MI, Rahman R, Colwell RR (1983). Ecological relationships between *Vibrio cholerae* and planktonic crustacean copepods. Appl Environ Microbiol.

[CR28] Jones MK, Oliver JD (2009). *Vibrio vulnificus*: disease and pathogenesis. Infect Immun.

[CR29] Kaneko T, Colwell RR (1975). Adsorption of *Vibrio parahaemolyticus* onto chitin and copepods. Appl Environ Microbiol.

[CR30] Klein SL, Lovell CR (2016). The hot oyster: levels of virulent *Vibrio parahaemolyticus* strains in individual oysters. FEMS Microbiol Ecol.

[CR31] Klein SL, West CK, Mejia DM, Lovell CR (2014). Genes similar to the *Vibrio parahaemolyticus* virulence related genes *tlh*, *tdh*, and *vscC2* occur in other *Vibrionaceae* species isolated from a pristine estuary. Appl Environ Microbiol.

[CR32] Letchumanan V, Kok-Gan C, Lee LH (2014). *Vibrio parahaemolyticus*: a review on the pathogenesis, prevalence, and molecular identification techniques. Front Microbiol.

[CR33] Liu D, Cole RA, Reeves PR (1996). An O-antigen processing function for Wzx (RfbX): a promising candidate for O-unit flippase. J Bacteriol.

[CR34] Lovell CR (2017). Ecological fitness and virulence features of *Vibrio parahaemolyticus* in estuarine environments. Appl Microbiol Biotechnol.

[CR35] Lui M, Zhang Y, Inouye M, Woychik NA (2008). Bacterial addiction module toxin Doc inhibits translation elongation through its association with the 30S ribosomal subunit. PNAS.

[CR36] Makino K, Oshima K, Kurokawa K, Yokoyama K, Takayuki U, Tagomori K, Iijima Y, Najima M, Nakano M, Yamashita A, Kubota Y, Kimura S, Yasunaga T, Honda T, Shinagawa H, Hattori M, Iida T (2003). Genome sequence of *Vibrio parahaemolyticus*: a pathogenic mechanism distinct from that of *V. cholerae*. Lancet.

[CR37] McPherson VL, Watts JA, Simpson LM, Oliver JD (1991). Physiological effects of the lipopolysaccharide of *Vibrio vulnificus* on mice and rats. Microbios.

[CR38] Metzger LC, Blokesch M (2016). Regulation of competence-mediated horizontal gene transfer in the natural habitat of *Vibrio cholerae*. Curr Opin Microbiol.

[CR39] Minato Y, Ghosh A, Faulkner WJ, Lind EJ (2013). Na+/H+ antiporter is essential for *Yersinia pestis* virulence. Infect Immun.

[CR40] Nalin DR, Daya V, Reid A, Levine MM, Cisneros L (1979). Adsorption and growth of *Vibrio chole*rae on chitin. Infec and Immun.

[CR41] O’Shea YA, Finnan S, Reen FJ, Morrissey JP, O’Gara F, Boyd EF (2004). The *Vibrio* seventh pandemic island-II is a 269 kb genomic island present in *Vibrio cholerae* El Tor and O139 serogroup isolates that shows homology to a 434 kb genomic island in *V. vulnificus*. Microbiol.

[CR42] Overbeek R, Begley T, Butler RM, Choudhuri JV, Chuang HY (2005). The subsystems approach to genome annotation and its use in the project to annotate 1000 genomes. Nucleic Acids Res.

[CR43] Overbeek R, Olson R, Pusch GD, Olsen GJ, Davis JJ, Disz T, Edwards RA, Gerdes S, Parrello B, Shukla M, Vonstein V, Wattam AR, Xia F, Stevens R (2014). The SEED and the Rapid Annotation of microbial genomesusing Subsystems Technology (RAST). Nucleic Acids Res.

[CR44] Park KS, Ono T, Rokuda M, Jang MH, Okada K, Iida T, Honda T (2004). Functional characterization of two type III secretion systems of *Vibrio parahaemolyticus*. Infect Immun.

[CR45] Quirke AM, Reen FJ, Boyd EF (2006). Genomic island identification in *Vibrio vulnificus* reveals significant genome plasticity in this human pathogen. Bioinformatics.

[CR46] Scallan E, Hoekstra RM, Angulo FJ, Tauxe RV, Widdowson MA, Roy SL, Jones JL, Griffin PM (2011). Foodborne illness acquired in the United States—major pathogens. Emerg Infect Dis.

[CR47] Schmidt H, Hensel M (2004). Pathogenicity islands in bacterial pathogenesis. Clin Microbiol Rev.

[CR48] Sugiyama T, Iida T, Izutsu K, Park KS, Honda T (2008). Precise region and the character of the pathogenicity island in clinical *Vibrio parahaemolyticus* strains. J Bacteriol.

[CR49] Tamplin ML, Gauzens AL, Huq A, Sack DA, Colwell RR (1990). Attachment of *Vibrio cholerae* serogroup O1 to zooplankton and phytoplankton of Bangladesh waters. Appl Environ Microbiol.

[CR50] Van Melderen L, Saavedra DM (2009). Bacterial toxin–antitoxin systems: more than just selfish entities?. PLoS Genet.

[CR51] Vimont S, Berche P (2000). NhaA, a Na^+^/H^+^ antiporter involved in environmental survival of *Vibrio cholerae*. J Bacteriol.

[CR52] Wang H, Wong MML, O’Toole D, Mak MMH, Wu RSS, Kong RYC (2006). Identification of a DNA methyltransferase gene carried on a pathogenicity island-like element (VPAI) in *Vibrio parahaemolyticus* and its prevalence among clinical and environmental isolates. Appl Environ Microbiol.

[CR53] Xu F, Gonzalez-Escalona N, Drees KP, Sebra RP, Cooper VS, Jones SH, Whistler CA (2017). Parallel evolution of two clades of an Altantic-endemic pathogenic lineage of *Vibrio parahaemolyticus* by independent acquisition of related pathogenicity islands. Appl Environ Microbiol.

[CR54] Yamamoto K, Wright AC, Kaper JB, Morris JG (1990). The cytolysin gene of *Vibrio vulnificus*: sequence and relationship to the *Vibrio cholerae* El Tor hemolysin gene. Infect Immun.

[CR55] Yanagihara I, Nakahira K, Yamane T, Kaieda S, Mayanagi K, Hamada D, Fukui T, Ohnishi K, Kajiyama S, Toshiyuki S, Sato M, Ikegami T, Ikeguchi M, Honda T, Hashimoto H (2010). Structure and functional characterization of *Vibrio parahaemolyticus* thermostable direct hemolysin. J Biol Chem.

